# Aluminum-Foil/Polyester Core-Spun Yarns Conductive Fabric Enabling High Electromagnetic Interference Shielding

**DOI:** 10.3390/polym18010145

**Published:** 2026-01-05

**Authors:** Yanyan Sun, Xiaoyu Han, Kun Zhao, Weili Zhao, Zhitong He, Zhengyang He, Yingtie Mo, Changliu Chu, Toshiaki Natsuki, Jun Natsuki

**Affiliations:** 1 School of Textile and Garment, Anhui Polytechnic University, Wuhu 241000, China; 2 Interdisciplinary Graduate School of Science and Technology, Shinshu University, Ueda 386-8567, Japan; 3 Chery Automobile Co., Ltd., Wuhu 241009, China; 4 Institute for Fiber Engineering and Science (IFES), Research Cluster for Social Implementation, Shinshu University, Ueda 390-8621, Japan; 5 Faculty of Textile Science and Technology, Shinshu University, 3-15-1 Tokida, Ueda 386-8567, Japan

**Keywords:** aluminum-foil wire, core-spun yarn, conductive fabric, electromagnetic interference shielding, mechanical durability, acid and alkali resistance

## Abstract

With the rapid advancement of modern electronic devices and wireless communication systems, electromagnetic pollution has become a prominent issue, prompting the development of high-performance electromagnetic interference (EMI) shielding materials. Although traditional metal shielding materials exhibit excellent conductivity, there are many limitations such as high weight, poor flexibility, susceptibility to corrosion, and high cost. To overcome these challenges, in this study, we design and fabricate core-spun yarns using polyester filaments as the core and an aluminum-foil-wrapped layer as the conductive outer component, and further weave them into three conductive fabrics with different structural parameters. Through systematic investigation of their surface morphology, air permeability, electrical properties, and EMI shielding performance, DT5W27 demonstrates optimal overall performance: electrical conductivity of 2722.64 S·m^−1^, shielding effectiveness of 37.29 dB, and electromagnetic wave attenuation rate of 99.99%. Specifically, even after 100 bending, twisting cycles, and exposure to solutions with pH values ranging from 3 to 9, the fabric maintains high shielding performance. The fabrication process is facile and low cost, and these composites have good flexibility, outstanding EMI shielding performance, exceptional mechanical durability, and chemical stability. These advantages make them have broad application potential in protective clothing and lightweight shielding materials.

## 1. Introduction

With the increasing popularity of modern electronic devices and wireless communication systems, imperceptible electromagnetic pollution has attracted considerable attention, which not only affects the normal operation of electronic systems, but also poses a potential threat to human health and environment [1,2]. Consequently, the development of high-performance EMI shielding materials can not only eliminate the harm of electromagnetic waves to human body and equipment but also ensure the reliability of electronic devices in complex electromagnetic environments and effectively inhibit unnecessary electromagnetic radiation, which is of great significance for communication industries, aerospace, defense, consumer electronics, and other fields [3,4,5,6].

EMI shielding refers to the physical phenomenon of attenuating electromagnetic field propagation through the deployment of conductive barriers [7,8]. In practice, the main purposes of EMI shielding are to confine electromagnetic energy emanating from the area that needs to be protected and to impede external electromagnetic radiation from entering the protected area [9]. The effectiveness of EMI shielding is determined by the material properties, geometric configuration, and frequency response characteristics of conductive barriers, which collectively mitigate both near-field inductive couplings and far-field electromagnetic wave transmissions [10,11]. Traditional metal plates, sheets, and screens made of copper, iron, nickel, or aluminum alloys have been widely used in the field of EMI shielding because of their exceptionally high electrical conductivity, which effectively reflects incident electromagnetic waves [12]. However, their inherent limitations, including rigidity, heavyweight, susceptibility to corrosion, and high processing costs, restrict their applications in flexible and lightweight scenarios [13,14].

In recent years, conductive textiles have emerged as promising alternatives, offering advantages such as flexibility, softness, porous structures, and ease of processing [15,16,17]. Numerous research efforts have focused on coating conductive materials, such as graphene, conductive polymers, MXene, etc., onto textile substrates (from fiber to yarn and then fabric) [18,19,20,21,22]. Wang et al. [23] adopted a dip-coating approach to fabricate MXene-decorated polyester textiles, which exhibited electrical conductivity of ~1000 S·m^−1^ in conjunction with EMI shielding effectiveness (SE) of ~90 dB at a thickness of 1.3 mm. Zhang et al. [24] employed layer-by-layer assembled technology and the dip-coating method to prepare EMI shielding fabric with an average SE value of about 33 dB. Akram et al. [25] performed electroless plating of silver on polyaniline/aminated graphene oxide-based fabric to obtain multilayer-structured textile that showed the highest shielding effectiveness of about 93 dB with 1.5 mm thickness. Duan et al. [26] prepared a carbon fiber fabric/graphene oxide/Fe_3_O_4_/epoxy composite with EMI SE up to 32.9 dB through the combined technology of electrohydrodynamic atomization deposition, layer-by-layer assembly, and heat-pressing. However, despite the recent advancements in conductive textile research, substantial challenges persist in achieving a robust interfacial adhesion between conductive materials and textile substrates, as well as enhancing the comprehensive performance of conductive fabrics.

Herein, composite conductive yarns with a stable core-wrapped structure are designed and fabricated by innovatively wrapping aluminum-foil wire around polyester filaments. These yarns are further woven into three aluminum-foil wire conductive fabrics with different densities (ST12W11, DT7W9, and DT5W27, S denotes a single yarn, D denotes double yarns plied together, while T and W represent the warp and weft directions, respectively, and the numerical values correspond to the respective warp and weft densities). The morphology, electrical properties, air permeability, and EMI shielding behavior and mechanism of these conductive fabrics in the microwave range (8.2–12.4 GHz) are systematically investigated. Furthermore, the durability of their shielding performance is rigorously evaluated under cyclic bending, twisting, and various pH conditions. This work provides a feasible and scalable route for developing high-performance, flexible, and environment-resistant textile-based shielding materials, demonstrating great potential for applications in wearable electronics, personal protective equipment, and lightweight EMI shielding packages.

## 2. Experimental Section

### 2.1. Preparation of Aluminum-Foil/Polyester Core-Spun Yarns

In the spinning process, the polyester filament bundle was employed as the core layer. Firstly, the material was subjected to positive feed rollers in order to undergo a specific drafting process. Subsequently, the polyester filament bundle, which had been previously prepared, was passed through the central tube of a hollow spindle. Meanwhile, the aluminum-foil yarn (purity > 99.0%) with width of 280 μm and thickness of 18 μm (purchased from Dongguan Chenghuigu Electronics Co., Ltd., Dongguan, China), which was wound around the hollow spindle as the covering layer, was unwound from the spindle tube. Subsequently, the unwound aluminum-foil yarn was then combined with the stretched polyester filament bundle. During the process of hollow-spindle spinning, the aluminum-foil yarn followed a helical path around the polyester bundle, thereby forming a compact core-sheath structure. In this configuration, the geometrical confinement and close contact between the sheath and the core generated a stable mechanical interlocking effect, which effectively restricted relative motion and prevented slippage of the aluminum foil on the polyester filaments. Through this combination, a pre-structured yarn segment was formed, in which the aluminum-foil yarn was wrapped around the polyester filament bundle. Finally, this pre-structured yarn segment was drawn out from the front end of the spindle, forming aluminum-foil/polyester core-spun yarns.

### 2.2. Fabrication of Aluminum-Foil/Polyester Core-Spun Yarns Conductive Fabrics

Aluminum-foil/polyester core-spun yarns were used as both warp and weft in a plain-weave configuration. The fabric architecture was systematically adjusted by employing steel reeds with varying counts and regulating the number of warp and weft yarns to achieve precise control over structural parameters. Using these settings, three woven fabrics with distinct configurations and warp/weft densities were fabricated on a sampling loom and designated as ST12W11, DT7W9, and DT5W27.

### 2.3. Characterizations

The surface micromorphology of aluminum-foil/polyester conductive fabrics was observed by a field-emission scanning electron microscope (SEM, CIQTEK SEM3200, CIQTEK Co., Ltd., Hefei, China) at 5 kV. A four-point-probe resistance instrument (RTS-8, Four Probe Tech. Co., Ltd., Guangzhou, China) was utilized to record the surface resistance of the conductive fabrics. A vector network analyzer (P5004A, Keysight Technologies, Santa Rosa, CA, USA) was employed to evaluate the EMI SE of aluminum-foil/polyester conductive fabrics within the frequency range of 8.2–12.4 GHz. The surface resistance and EMI SE tests were repeated five times, and the average values were calculated. SE can be expressed as follows [27,28]:

(1) $$R={\left|S_{11}\right|}^{2},T={\left|S_{21}\right|}^{2},A=1-R-T$$

(2) $${\mathrm{SE}}_{R}=-10\log(1-R),{\mathrm{SE}}_{A}=-10\log(T/(1-R))$$

(3) $$\mathrm{SE}={\mathrm{SE}}_{R}+{\mathrm{SE}}_{A}=-10\log T$$

where R, T, and A represent the reflection, transmission, and absorption coefficient, respectively.

R, T, and A are determined by scattering parameters (S_11_ and S_21_). SE_R_ denotes reflective electromagnetic shielding effectiveness; SE_A_ refers to absorbing electromagnetic shielding effectiveness, and the EMI SE is the sum of SE_R_ and SE_A_.

## 3. Results and Discussion

### 3.1. Fabrication and Characterization of Aluminum-Foil/Polyester Conductive Fabrics

**Figure 1:**
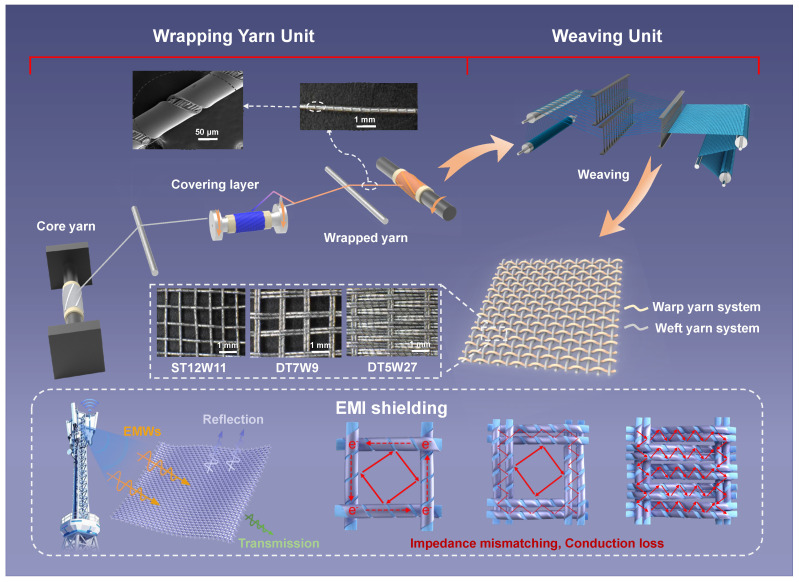
Manufacturing and structure schematic diagrams of aluminum-foil/polyester conductive fabrics. Polyester filament bundle as core is wrapped with aluminum-foil yarn to form core-spun yarn, which is woven into plain fabrics on the weaving machine. The colorful arrows indicate incident (yellow), reflected (purple), transmitted (green), and multiply reflected (red) electromagnetic waves during EMI shielding.

Figure 1 illustrates the preparation process of aluminum-foil/polyester conductive fabrics involving two interconnected units: the wrapping yarn unit and the weaving unit. In the wrapping yarn unit, polyester filament undergoes drafting and is wrapped by aluminum-foil yarn via a wrapping system, forming aluminum-foil/polyester core-spun yarns, which are then collected by a roll-up system. The weaving unit utilizes the core-spun yarns as the warp and weft yarns to form three different structures and densities of aluminum-foil/polyester conductive fabrics (ST12W11, DT7W9, DT5W27). Table 1 presents technological parameters of three aluminum-foil/polyester conductive fabrics. It can be clearly found that aluminum-foil/polyester conductive fabrics prepared by adjusting the number of warp and weft yarns show significant differences in weight, thickness, and density. The thickness of ST12W11 is 0.340 mm, while that of DT7W9 reaches 0.358 mm, and that of DT5W27 is 1.034 mm. This thickness variation is closely related to the fabric architecture: ST12W11 is woven with single core-spun yarns at relatively low weft density, whereas DT7W9 and DT5W27 adopt double core-spun yarns. In particular, DT5W27 combines a double-yarn configuration with the highest weft density, leading to a more compact stacked structure and greater through-thickness accumulation of conductive yarns, thereby resulting in its substantially larger thickness. These measurements are crucial for the EMI shielding performance.

**Figure 2:**
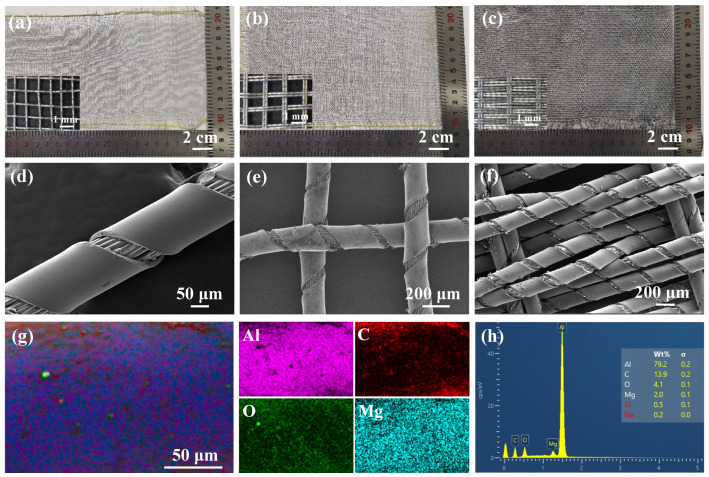
Optical photographs of aluminum-foil/polyester conductive fabrics with different weave structures: (**a**) ST12W11, (**b**) DT7W9, and (**c**) DT5W27. (**d**) SEM image of the aluminum-foil/polyester core-spun yarn. SEM micrographs of (**e**) ST12W11 and (**f**) DT7W9. (**g**) EDS elemental maps of the aluminum-foil/polyester yarn. (**h**) Elemental distribution of the aluminum-foil/polyester yarn.

As illustrated in Figure 2a–c, three aluminum-foil/polyester conductive fabrics were examined using optical photographs. The macroscopic appearance and the corresponding optical microscopy insets reveal distinct surface morphologies governed by the yarn type and weave parameters. For ST12W11 (Figure 2a), the single aluminum-foil/polyester core-spun yarns yield a uniform plain-weave texture; both the macroscopic image and the microscopy inset clearly show discernible inter-yarn gaps. DT7W9 (Figure 2b), fabricated using two-ply yarns in both warp and weft, exhibits a more compact surface at the macroscopic scale, which is further corroborated by the inset showing reduced inter-yarn spacing and lower apparent porosity. In DT5W27 (Figure 2c), the substantially increased weft density results in the highest structural compactness, evidenced by a tightly packed yarn arrangement and minimal inter-yarn gaps in the microscopy inset. These observations are consistent with the structural parameters summarized in Table 1, confirming that the yarn configuration and warp/weft density collectively determine the fabric compactness.

The microstructure of the aluminum-foil/polyester core-spun yarn is revealed in Figure 2d. The yarn consists of a polyester filament core that serves as the structural support, helically wrapped by an aluminum-foil yarn forming the outer conductive layer. The aluminum-foil wrapping remains continuous and adherent, producing a smooth metallic surface without fiber protrusion or foil fracture. The polyester filaments are arranged in an orderly twisted alignment, and no interfacial voids are observed between the aluminum-foil yarn and the polyester core, indicating an integrated core-spun structure.

When these core-spun yarns are interwoven to form fabrics (Figure 2e,f), they preserve their core-spun morphology at the fabric scale. In ST12W11, single core-spun yarns interlace into a regular plain-weave grid with a clearly identifiable aluminum-foil wrapping around the polyester core. In DT7W9, the use of two-ply yarns results in a more compact yarn-bundle configuration and a denser woven structure, thereby reducing surface porosity and enhancing microstructural uniformity. These observations further confirm that yarn type and wrapping configuration strongly influence the macroscopic compactness of the conductive fabrics.

The elemental distributions of the aluminum-foil yarn are shown in Figure 2g,h. The elements Al, C, O, and Mg were detected on the yarn surface, and all of them are uniformly distributed. In addition, the atomic ratios of Al/C/O/Mg were approximately 79.2%/13.9%/4.1%/2.0%, indicating that Al is the dominant element on the yarn surface.

**Figure 3:**
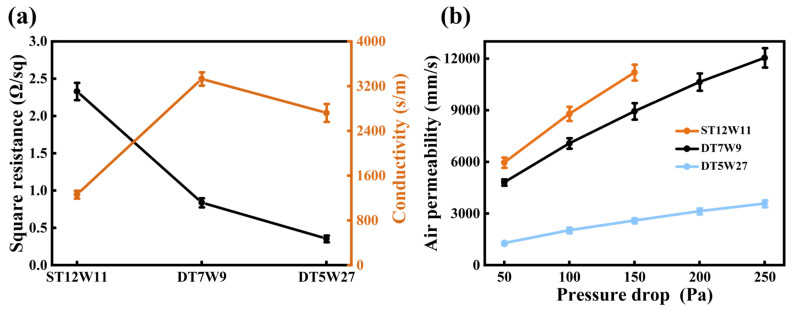
(**a**) Electrical resistance, conductivity, and (**b**) air permeability under various pressure differential of aluminum-foil/polyester conductive fabrics.

Figure 3a summarizes the square resistance and conductivity of aluminum-foil/polyester conductive fabrics with different structures. Specifically, ST12W11 demonstrates a square resistance of 2.33 Ω·sq^−1^, with the corresponding electrical conductivity of 1262.63 S·m^−1^. The square resistance of DT7W9 decreases significantly to 0.84 Ω·sq^−1^, while the electrical conductivity increases substantially to 3329.97 S·m^−1^, indicating superior conductive behavior. Compared with DT7W9, the square resistance of DT5W27 further reduces to 0.36 Ω·sq^−1^ due to more conductive yarns per unit area in DT5W27, forming more conductive pathways. Conversely, the electrical conductivity of DT5W27 declines to 2722.64 S·m^−1^. This phenomenon may arise from the uneven distribution of conductive pathways caused by the excessive compactness of the fabric structure. The compact DT5W27 structure may induce local folding, wrinkling, and partial damage of the aluminum-foil wrapping, and reduce the effective contact area between adjacent yarns, thereby interrupting continuous conductive pathways and increasing contact resistance. These results confirm that the structural differences of aluminum-foil/polyester conductive fabrics have a significant impact on their electrical properties [29].

The air permeability of aluminum-foil/polyester conductive fabrics under the pressure differential ranging from 50 to 250 Pa is depicted in Figure 3b. It is evident that all three fabric specimens exhibit a consistent upward trend in air permeability as the pressure differential increases. Notably, the increasing trends of air permeability for ST12W11 and DT7W9 are relatively similar. ST12W11 achieves an air permeability of 11,200 mm/s under the pressure differential of 150 Pa, while DT7W9 reaches approximately 12,050 mm/s at 250 Pa. On the contrary, DT5W27 demonstrates significantly lower overall air permeability than the other two fabrics. Under identical pressure differentials, it shows the weakest air permeability, measuring only about 3600 mm/s at 250 Pa.

### 3.2. EMI Shielding Performance and Mechanism of Aluminum-Foil/Polyester Conductive Fabrics

**Figure 4:**
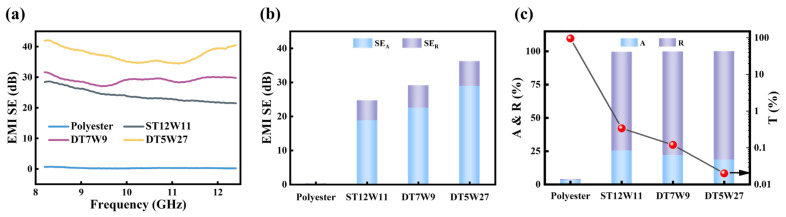
(**a**) EMI shielding performance of polyester fabric and aluminum-foil/polyester conductive fabrics under frequency range of 8.2–12.4 GHz. (**b**) SE_R_ and SE_A_ and (**c**) power coefficients (A, R, T) of polyester fabric and aluminum-foil/polyester conductive fabrics at 10 GHz.

Figure 4a presents the EMI SE of polyester fabrics and aluminum-foil/polyester conductive fabrics showing relatively stable throughout the frequency range of 8.2–12.4 GHz, exhibiting only minor fluctuations. Overall, the EMI SE of each sample is relatively stable with frequency, with only slight fluctuations. Due to its electrical insulation, the polyester fabric shows very limited electromagnetic shielding capability, as reflected by its low average EMI SE of only 0.32 dB. In contrast, the aluminum-foil/polyester conductive fabrics demonstrate remarkably enhanced shielding properties. Among them, DT5W27 demonstrates an average EMI SE of 37.29 dB, translating to an effective shielding efficiency of 99.99%. This substantial attenuation confirms the critical role of conductive pathways formed by the aluminum-foil in facilitating strong reflection and absorption of electromagnetic waves. It is worth noting that the EMI shielding performance of aluminum-foil/polyester conductive fabrics with varied structures (ST12W11, DT7W9, DT5W27) meet the industrial application standard for EMI shielding materials (20 dB). Moreover, the EMI shielding performance can be significantly regulated by adjusting the preparation parameters of aluminum-foil/polyester conductive fabrics.

Collectively, the EMI shielding performance can be systematically modulated by tailoring the structural and processing parameters of the aluminum-foil/polyester conductive fabrics, such as weave pattern, wire density, and alignment. This tunability highlights the versatility of the fabrication strategy and enables the optimization of shielding properties for diverse operational environments.

To systematically elucidate the EMI shielding mechanism, SE_R_, SE_A_, and the corresponding power coefficients (A, R, T) are calculated in accordance with Equations (1)–(3) in the Experimental Section. As shown in Figure 4b, the polyester fabric exhibits negligible shielding capability. In contrast, DT5W27 has an SE_A_ of 28.96 dB, whereas its SE_R_ measures only 7.29 dB. This dominance of SE_A_ over SE_R_ is also consistently observed in the samples ST12W11 and DT7W9.

Notably, the reflection coefficient R of DT5W27 reaches 81.34%, while the corresponding absorption coefficient A is 18.64%, and the transmission T is 0.02% (Figure 4c). The relatively high R indicates a pronounced impedance mismatch at the fabric–air interface and strong reflection from surface of aluminum-foil. The reason for the different trend between ‘SEA>SER’ and ‘A<R’ is that SEA calculates the shielding effectiveness contributed by the absorption of electromagnetic waves after they enter into the material, without considering the electromagnetic waves reflected at the surface; whereas A represents the percentage of absorbed electromagnetic energy relative to the total incident electromagnetic energy. In this context, aluminum-foil/polyester conductive fabrics due to their excellent electrical conductivity cause most electromagnetic waves to be reflected at their surface. The portion of electromagnetic waves that do penetrate into the aluminum-foil/polyester conductive fabrics interior are absorbed and dissipated through mechanisms such as ohmic losses and multiple internal reflections.

Table 2 compares the EMI shielding performance of aluminum-foil/polyester conductive fabrics developed in this work with previously reported materials. Aluminum-foil wire conductive fabrics exhibit superior EMI shielding ability comparable to that of most composite- [30,31,32,33,34], film- [35,36], foam- [37], and fabric-based [38,39] EMI shielding materials. Specifically, they show a compelling combination of properties, achieving an EMI SE of 37.29 dB at a thickness of 1.034 mm, which is much lower than other metal/graphene/MXene-based composites or foams [30,32,33,37], and an electrical conductivity of 2722.64 S·m^−1^, which is higher than other composites and fabrics [32,33,34,38], thus demonstrating competitive SE. These characteristics underscore the potential of aluminum-foil/polyester conductive fabrics as practical and efficient candidates for EMI shielding applications.

**Figure 5:**
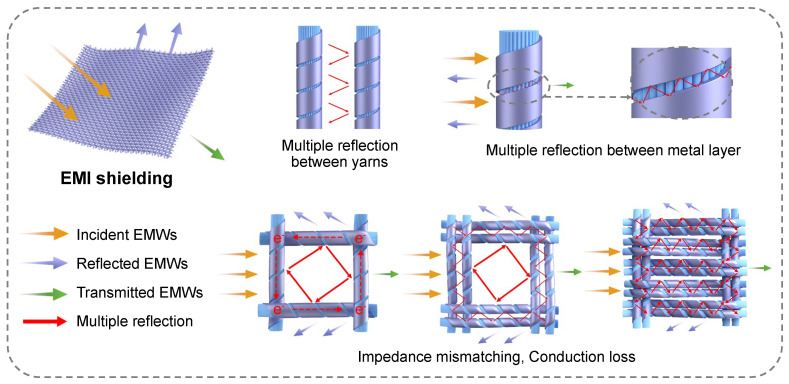
Schematic illustration of the EMI shielding mechanism for aluminum-foil/polyester conductive fabrics.

As illustrated in Figure 5, the EMI shielding mechanism of aluminum-foil/polyester conductive fabrics is attributed to the synergistic effect of reflection and absorption, with the compactness of the fabric structure playing a critical role in enhancing shielding performance. When incident electromagnetic waves hit aluminum-foil/polyester conductive fabrics, a large part of them are immediately reflected due to the impedance mismatch at the air–metal interface. For those that penetrate the initial reflective barrier, the interleaved layered structure of aluminum-foil/polyester conductive fabrics induces multiple path reflection in the gap between fibers and metal, which prolongs the energy dissipation process and effectively reduces the intensity of electromagnetic waves. Concurrently, electromagnetic coupling excites collective oscillation of free electrons within the aluminum-foil, leading to conduction loss through electron collisions and frictional heat conversion, which enhances the absorption of electromagnetic waves. It is worth noting that the polyester filament serves as framework, ensuring the orderly arrangement of aluminum-foil yarns and the continuity of conductive network and preventing the failure caused by a loose structure.

As the fabric structure becomes denser, the pores between aluminum-foil conductive yarns decrease, which further enhances the performance of EMI shielding. Firstly, the shortened yarn spacing and compacted layer structure increase the density and complexity of reflection paths, intensifying the reflection loss caused by impedance mismatch. Secondly, the closer contact between aluminum-foil conductive yarns enhances the integrity of conductive networks, facilitating longer electron migration paths and more frequent collisions, thereby significantly improving the absorption efficiency of electromagnetic waves. Additionally, the tight weaving of yarns reinforces the mechanical stability of fabric, reducing the gap caused by deformation and further suppressing transmitted electromagnetic waves.

**Figure 6:**
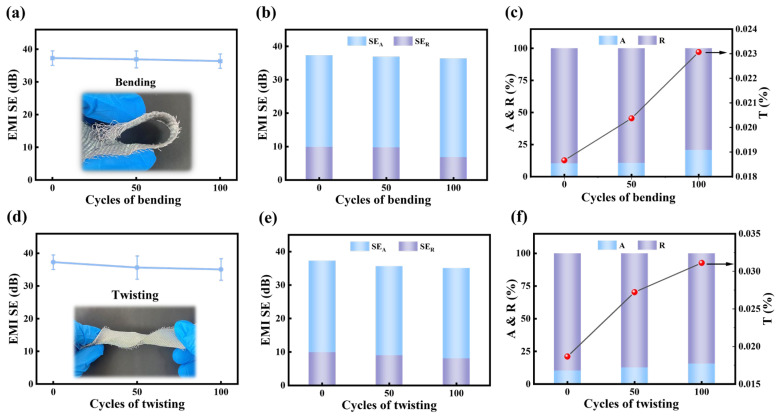
Variation of the EMI shielding performance of DT5W27 under mechanical deformation: (**a**) EMI SE, (**b**) SE_A_, SE_R_, and (**c**) power coefficients (A, R, T) of DT5W27 with bending cycles. (**d**) EMI SE, (**e**) SE_A_, SE_R_, and (**f**) power coefficients (A, R, T) of DT5W27 with twisting cycles.

To evaluate the durability and stability of aluminum-foil/polyester conductive fabrics in practical applications, their EMI shielding performance is systematically investigated under different bending and twisting cycles. As shown in Figure 6a, after 100 cycles of bending, the EMI SE of DT5W27 remains nearly constant at around 37 dB, indicating its excellent mechanical stability and structural integrity of the conductive network. Further analysis reveals that the shielding effectiveness is primarily contributed by SE_A_, while the contribution of SE_R_ is relatively minor, as illustrated in Figure 6b. The corresponding power coefficients (Figure 6c) show that the T value of DT5W27 is only 0.023% after 100 bending cycles, demonstrating that the fabric effectively blocks the penetration of electromagnetic waves. Meanwhile, the proportions of R and A remain nearly unchanged after 50 cycles of bending, further confirming the stable shielding performance of aluminum-foil/polyester conductive fabrics during bending.

Similarly, after different twisting cycles, the EMI SE of DT5W27 exhibits only a slight decrease, maintaining a high level of approximately 35 to 37 dB, which demonstrates remarkable torsional durability (Figure 6d). Consistent with the bending results, SE_A_ dominates the shielding mechanism, while transmission remains negligible (Figure 6e,f). These results indicate that aluminum-foil/polyester conductive fabrics retain prominent EMI shielding performance and mechanical reliability under repeated bending and twisting, making them highly suitable for flexible wearable devices and other demanding applications.

**Figure 7:**
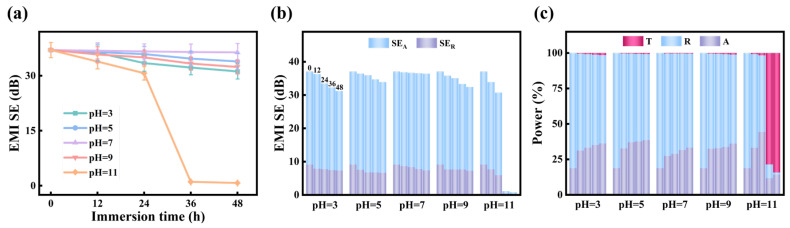
Evolution of the EMI shielding performance of DT5W27 as functions of immersion time and pH: (**a**) EMI SE, (**b**) SE_A_, SE_R_, and (**c**) the corresponding power coefficients (A, R, T).

Figure 7a presents the effect of pH on EMI SE of aluminum-foil/polyester conductive fabrics. After being immersed in solutions with pH values of 3, 5, 7, and 9 for 0, 12, 24, 36, and 48 h, the EMI SE of DT5W27 remains stable at approximately 32 to 35 dB, which indicates prominent chemical stability under mildly acidic, neutral, and weakly alkaline conditions. In contrast, a drastic deterioration in EMI SE is observed in the solution with pH 11, where the shielding performance drops sharply after 24 h immersion and eventually decreases to nearly 0 dB after 36 h. This is mainly because strong alkaline environments severely corrode aluminum-foil, destroying the conductive structure of fabrics and leading to the complete loss of shielding functionality. Overall, aluminum-foil/polyester conductive fabrics demonstrate excellent EMI shielding stability in a broad pH range (3–9), except for strongly alkaline conditions (pH 11).

To deeply analyze the shielding mechanism of aluminum-foil/polyester conductive fabrics under different pH conditions, the contributions of SE_A_ and SE_R_ to the total shielding effectiveness, as well as the power distribution of T, R, and A, are shown in Figure 7b,c. It can be clearly seen that absorption remains the dominant shielding mechanism across all pH values, while reflection contributes to a lesser extent. In the pH range from 3 to 9, both SE_A_ and SE_R_ exhibit only slight fluctuations with immersion time, confirming the structural stability of DT5W27 in mildly acidic, neutral, and weakly alkaline environments. However, at pH 11, both SE_A_ and SE_R_ decrease significantly with prolonged immersion time, which is consistent with the results observed in Figure 7a.

The corresponding power coefficients (Figure 7c) further validate these observations. Within the pH range of 3 to 9, the T value remains nearly zero, while A and R values maintain a stable ratio, suggesting reliable shielding performance in these pH environments. In contrast, at pH 11, the T value rapidly increases with immersion time, while the A and R values decrease sharply, indicating severe degradation of the conductive network within DT5W27 under strongly alkaline conditions. These results confirm that aluminum-foil/polyester conductive fabrics retain consistent EMI shielding functionality in a wide pH range, with absorption serving as the primary shielding mechanism, but undergo structural failure under strongly alkaline conditions.

**Figure 8:**
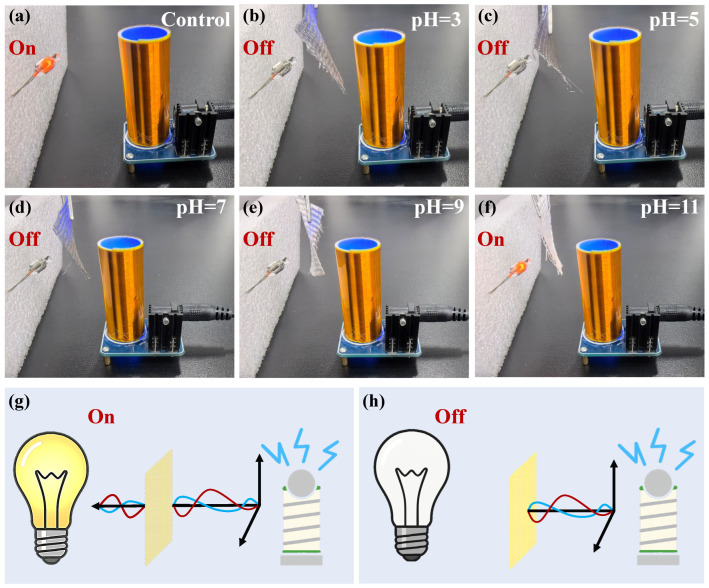
EMI shielding performance of DT5W27 conductive fabric under different pH conditions, demonstrated using a Tesla coil. (**a**) Control group (without fabric). (**b**–**f**) Shielding capability of the DT5W27 fabrics after being immersed in solutions with pH values of 3, 5, 7, 9, and 11 for 48 h. (**g**,**h**) Schematic diagram of small light bulb response under Tesla-coil excitation with different shielding fabrics.

A Tesla coil was used to visually evaluate the EMI shielding performance of DT5W27 after being immersed in solutions with different pH values for 48 h. As illustrated in Figure 8, when the Tesla coil was activated, a nearby fluorescent bulb emitted bright light under the influence of the induced electromagnetic field. Significant differences in shielding behavior were observed when the DT5W27 fabric exposed to various pH conditions were placed between the coil and the bulb. At pH levels of 3, 5, 7, and 9 (Figure 8b–e), the bulb remained completely unlit, indicating that the fabric effectively blocked electromagnetic radiation and maintained an uninterrupted conductive network. This result aligns with the stable EMI shielding effectiveness (32–35 dB) shown in Figure 7a, confirming the chemical stability of the aluminum-foil/polyester in mildly acidic, neutral, and weakly alkaline environments. In contrast, at pH 11 (Figure 8f), the bulb glowed brightly even with the fabric in place, revealing a severe decline in shielding performance. The pronounced alkaline corrosion and subsequent delamination of the aluminum-foil/polyester disrupted the conductive pathways, leading to an almost complete loss of electromagnetic attenuation. Thus, the Tesla coil experiment visually confirms that the DT5W27 conductive fabric retains excellent EMI shielding stability within the pH range of 3–9, whereas it suffers complete shielding failure under strong alkaline conditions (pH 11) due to chemical degradation of the metallic framework.

In this context, the EMI shielding application was further evaluated by examining the ability of the fabrics to inhibit electromagnetic waves from energizing an LED bulb, as illustrated in Figure 8g,h. When a fabric with weak EMI shielding capability was placed between the Tesla coil and the LED bulb, it allowed substantial electric field transmission, maintained field coupling with the bulb, and therefore kept it illuminated (Figure 8g). In contrast, when a fabric with strong EMI shielding performance was inserted, it effectively suppressed the incident electric field, disrupted the coupling, and caused the bulb to turn off (Figure 8h).

## 4. Conclusions

In summary, core-spun yarns have been developed using polyester filaments as the core and aluminum-foil wrapping as the conductive outer layer. Three types of conductive fabrics with different structural parameters (ST12W11, DT7W9, and DT5W27) were prepared by adjusting warp and weft densities. Among them, DT5W27 shows an electrical conductivity of 2722.64 S·m^−1^ and exhibits outstanding electromagnetic shielding properties, including average EMI SE of 37.29 dB in the frequency range of 8.2–12.4 GHz at a thickness of 1.034 mm and shielding efficiency of 99.99%. Notably, DT5W27 maintains high shielding performance after 100 bending cycles, repeated twisting, and exposure to environments with pH values ranging from 3 to 9, demonstrating prominent mechanical durability and chemical stability. In addition, the fabrics retain desirable air permeability and flexibility, making them more suitable for conformable and comfort-oriented shielding applications than traditional rigid metal-based shields.

Overall, the aluminum-foil/polyester conductive fabrics developed in this work can be fabricated via a simple, low-cost, and scalable spinning–weaving process, while delivering a favorable balance of lightweight characteristics and efficient EMI shielding. These attributes make them promising candidates for use in EMI shielding garments, personal protective clothing, and other textile-based protection systems that require both comfort and electromagnetic safety. Moreover, their combination of flexibility, formability, and high shielding effectiveness suggests great potential in lightweight shielding components for consumer electronics, flexible electronic enclosures, soft packaging of wireless devices, and other scenarios where reliable EMI mitigation and structural adaptability are simultaneously demanded.

## Figures and Tables

**Table 1:** Technological parameters of aluminum-foil/polyester conductive fabrics.

Fabric	Warp Yarn	Weft Yarn	Reed	Fabric Weight (g/m^2^)	Thickness (mm)	Warp Density (Ends/cm)	Weft Density (Picks/cm)
ST12W11	150D	150D	115#	121.70	0.340	11.8	10.7
DT7W9	150D × 2	150D × 2	70#	155.00	0.358	7.2	9.1
DT5W27	150D × 2	150D × 2	50#	288.85	1.034	5.4	27.0

**Table 2:** Comparison of EMI shielding material with previous reports in the literature.

Materials	Form	EMI SE (dB)	Thickness (mm)	Conductivity (S·m^−1^)	Ref.
Aluminum/polyester	Fabric	37.29	1.034	2722.64	This work
Al/Ag/Methyl-vinyl si-rubber	Composite	70	20	10,000	[30]
Graphene/cellulose	Composite	28.3	0.043	/	[31]
Ti_3_Si(Al)C_2_-Al_2_O_3_/SiC	Composite	42.1	3	666	[32]
Ti_3_SiC_2_/Al	Composite	39	3	853	[33]
Fe-SiC_f_/SiC	Composite	39.29	0.7	504	[34]
Graphene/AgNPs/aluminum	Film	92.29	0.0345	4431	[35]
Aluminum ion-MXene	Film	80	0.039	265,600	[36]
Al-Si8Cu3Fe+ Cenosphere	Foam	−56.68	2	/	[37]
MXene/CuS/cotton	Fabric	51.1	0.335	1353	[38]
Polyester/polyvinylidene fluoride/additives	Fabric	19	0.35	/	[39]

## Data Availability

The original contributions presented in this study are included in the article. Further inquiries can be directed to the corresponding authors.
